# Atherosclerotic Cardiovascular Diseases in Middle Delta of Egypt: A Systematic Analysis of Risk Factors Associated with the Rising Burden of the Disease

**DOI:** 10.5334/gh.1395

**Published:** 2025-02-03

**Authors:** Mohamed Khalfallah, Marwa Habib, Ahmed Mustafa Kishk, Baraka Saeed, Shreen Hemdan, Ahmad Eissa, Ahmed A. Aboomar, Rasha Youssef Hagag, Basma Elnagar

**Affiliations:** 1 Cardiovascular medicine department, faculty of medicine, Tanta University, Egypt; 2 Neurology medicine department, faculty of medicine, Tanta University, Egypt; 3 Family medicine and Community health department, United Arab Emirates; 4 Internal medicine department, faculty of medicine, Tanta University, Egypt

**Keywords:** Atherosclerotic cardiovascular diseases, risk factors, Egypt

## Abstract

**Background:**

The popularity of behavioural and metabolic risk factors associated with atherosclerotic cardiovascular diseases (ACVDs) has increased because of social progress, rapid economic development, population aging, and changes in social ideology. We aimed to perform a systematic analysis of risk factors associated with the rising rate of ACVDs in Egypt.

**Methods:**

This study was carried out on 1,700 participants. The patients were classified into two groups: group 1 included patients with ACVDs, and group 2 (control group) included healthy individuals. All data recorded included patients’ anthropometric measurements, and laboratory and clinical examinations were collected.

**Results:**

The rising burden of ACVDs in Egypt was caused by a variety of risk factors, including diabetes mellitus, smoking, hypertension, dyslipidemia, obesity, and a lack of physical activity. The dominant risk factors recognized through multivariate regression analysis were the existence of metabolic syndrome (OR = 1.463; 95% CI, 1.056–2.026; P = 0.022), increased psychosocial stress among the patients (OR = 1.404; 95% CI, 1.008–1.953; P = 0.044), excessive consumption of high-fat, processed, and fast food (OR = 1.964; 95% CI, 1.489–2.590; P = 0.001), and decreased the income (OR = 1.865; 95% CI, 1.454–2.391; P = 0.001).

**Conclusions:**

Patients who suffer from uncontrolled diabetes, dyslipidemia, and metabolic syndrome are the most liable to have ACVDs. Psychosocial stress and the excessive intake of processed, high-fat, and fast food are augmenting leading risk features, especially in low-income populations.

## Introduction

Coronary artery disease (CAD) is a rapidly expanding and severe public health issue. The global incidence of IHD is increasing. It is anticipated that the current prevalence rate of 1,655 per 100,000 population in 2017 is expected to exceed 1,845 by the year 2030 (1). In addition, in 2017 IHD was responsible for the mortality of almost nine million people, making it the top cause of death globally. IHD has held its top spot for almost two decades (1). According to the most recent WHO statistics, coronary heart disease fatalities account for 32.40% of all deaths in Egypt, ranking the nation as the 15^th^ highest in the world (2).

As per the national hypertension initiative, the overall adjusted occurrence of CAD was 8.3%. The death rate related to age in Egypt was 186.36 per 100,000, according to the World Health Organization’s ranking, which placed the country at number 23 in the globe. Additionally, 23% of the total deaths were ascribed to CAD (3). Additionally, stroke deaths increased from 41.69 deaths in 2000 to 43.84 deaths in 2019 per 100,000 Egyptian population (4).

Atherosclerosis is a condition characterized by the accumulation of fatty substances, including cellular debris, cholesterol, and calcium, along the inner surface of the walls of the arteries. Plaques, which are tenacious yellowish deposits, are the most prevalent cause of chronic arterial occlusive disease and may lead to the narrowing of the arteries (5). Atherosclerotic disease is characterized by a complex and progressive pathophysiology. Silently, it develops in various vascular territories prior to the occurrence of an ischemic event or the functional relevance of a stenosis (6).

Smoking, high blood pressure, a high-sodium diet, kidney dysfunction, a high body mass index (BMI), high low-density lipoprotein (LDL) cholesterol, and high fasting plasma glucose (FPG) are among the behavioural and metabolic risk factors of atherosclerotic cardiovascular diseases (ACVDs). This has resulted in a substantial rise in morbidity and mortality due to cerebrovascular diseases and ACVDs (7). The analysis of the influence of metabolic risk factors on the morbidity and mortality of ACVDs over time is warranted. It was observed that a significant increase in the occurrence of metabolic risk factors hindered the control of morbidity and mortality due to a variety of ACVDs, including ischaemic heart diseases (IHD), and cerebrovascular diseases (8,9).

The increasing demand for prevention and treatment of IHD and other ACVDs is becoming increasingly urgent in developing countries, which make up over 80% of the global population. In these countries, the disease landscape is transitioning from infectious diseases to non-communicable diseases (NCDs) (10). As a result, public health experts and policymakers can greatly benefit from a comprehensive and current understanding of the overall burden of IHD, its features regarding epidemiology, and any associated environmental, metabolic, and behavioural risk factors. This knowledge can be used to develop and implement targeted programs that are designed to prevent and manage the IHD burden (11). In Egypt, the epidemiological characteristics and risk factors of ACVDs have not been extensively documented on a global scale, to the best of our knowledge. Consequently, this clinical study was initiated to perform a systematic analysis of risk factors associated with the rising burden of cardiovascular diseases in middle delta of Egypt.

## Patients and Methods

### Study design

The participants were selected from the outpatient clinic visitors at the cardiovascular, internal medicine, and neurology department, Tanta University Hospital. The study was designed to collect a convenient sample of the participants during the period from June 2021 to June 2023. An informed written consent for authorizing the collection of this data was collected from all participants. The research was consented to by the Ethical Committee of the Faculty of Medicine, Tanta University in concordance with the statement of Helsinki II principles with approval code 36264PR540/2/24. The correlation between traditional risk factors and CVD attenuated with aging; patients above 70 years were excluded to demonstrate the preventive risk factor effects. All enrolled 1700 participants were classified into two groups: Group I included 840 patients with a confirmed diagnosis of cardiovascular diseases including a former diagnosis of cerebrovascular diseases (history of ischemic or hemorrhagic stroke, transient ischemic attack, and carotid stenosis or surgery), IHD (history of myocardial infarction, angina or cardiomyopathy with documented coronary arteries stenosis, coronary revascularization), and peripheral arterial diseases (documented distal arteries stenosis, intermittent claudication, diabetic foot, previous vascular revascularization, and amputation), and Group II (control group) involved 860 individuals gathered among the relatives of the patients and the visitors of outpatient clinics. The control group was subjected to clinical history and physical examination in addition to stress ECG and carotid duplex and duplex of arterial system of lower limbs and coronary computed tomography as a screening for ACVDs before enrolling to exclude the existence of cardiovascular diseases (subclinical atherosclerosis was defined as the existence of a minimum one plaque that was demarcated as an increase in intima-media thickness more than 50% of the adjacent or a localized thickening over 1.5 mm, ankle-brachial index <0.90 and coronary artery calcium score ≥1) (12). The two groups were designed to be similar in age and sex distribution to exclude their effect as a risk factor on the final results.

### Clinical characteristics and risk factor history

We assembled a thorough comprehensive medical history from every patient, covering their medical history of chronic disease, e.g., diabetes mellitus (DM), hypertension, obstructive sleep apnoea, dyslipidaemia, atrial fibrillation, IHD, cerebrovascular diseases, peripheral arterial diseases, chronic kidney diseases, family history of IHD, obesity, smoking history, and medication used.

### Socioeconomic factors

Socioeconomic data such as marital status, level of education, residence, occupational status, and health insurance existence were collected. Furthermore, the presence of psychosocial stress and its degree was estimated by a questionnaire using a perceived stress scale (27–40: high perceived stress, 14–26: moderate stress, 0–13: low stress) (13). Questionnaires were collected from all participants for their detailed dietary habits and physical activity and analysed subsequently into adequate fruit/vegetable consumption ≤ 5 per portion of fruits/vegetables a day; excess use of salt diet >5 g sodium per day, which is equal to the consumption of more than one table salt (>1 teaspoon per day); excess sugar intake>50 gm per day, which is equal to 12 teaspoon (each teaspoon approximately contains 4 gm of sugar), for example, 330 ml of soda drink or juice equal to 35 gm of sugar; and high fast-food diet >2 times per week. Less than 75 minutes per week of vigorous-intensity exercise, 150 minutes per week of moderate intensity, or <10 minutes of any type of physical activity per day were considered inadequate physical activity (14,15).

### Physical and laboratory measurements

BMI was estimated by the formulation = Weight (kg)/(Height in meter2). According to the World Health Organization (WHO) criteria, BMI is classified into obese (≥30), overweight (25–29.9), normal (18.5–24.9), and underweight (<18.5) (15). Blood pressure was determined through the utilization of a calibrated digital sphygmomanometer in the vital room. Afterward, the measurement was repeated during the physical examination twice at least five minutes apart; the average of the measurements was recorded (16). A blood sample was gained from all participants from the antecubital vein for complete blood count, urea and creatinine, estimated glomerular filtration rate (e-GFR), thyroid stimulating hormone (TSH), fasting and two-hour postprandial blood glucose, complete lipid profile, uric acid, and C-reactive protein. Urine samples were collected for albuminuria. Full clinical examination involving echocardiography and twelve-lead surfaces ECG was performed in all patients.

### Statistical analysis

Statistical analysis was performed using SPSS 23, (SPSS Inc. Released 2015. IBM statistics for Windows, version 23, Armonk, NY; IBM Corp.). Qualitative variables were specified as frequency and percentage. The Chi-square test (χ2) was used to evaluate two qualitative parameters. Quantitative variables were specified as mean ± standard deviation. Independent samples t-test was used for evaluating quantitative variables between the two groups. P value <0.05 was considered statistically significant. Multivariate logistic regression analysis was achieved to assess the independent risk factors associated with the rising burden of ACVDs.

## Results

The aim of the current study was to evaluate the burden of cardiovascular diseases in middle delta of Egypt in relation to a variety of risk factors. By analysing 1,700 participants were classified into Group I consisted of 840 individuals with established diagnoses of cardiovascular diseases, and Group II consisted of 860 individuals as a healthy control group. The distribution of cardiovascular diseases among patients of Group I, we found that ischemic heart diseases, cerebrovascular diseases, and peripheral arterial diseases represented 71.3%, 24.2%, and 26.7%, respectively.

Despite the lack of differences in both age and sex among the two groups, Group I had a significantly higher prevalence of hypertension, smoking, diabetes mellitus, dyslipidemia, metabolic syndrome, and obesity. These findings demonstrated the major and basal features as well as their risk reasons preceding ACVDs, which illustrated in Table 1. Moreover, Table 2 distinguished the remarkable socioeconomic varieties between the participants. The psychosocial stress highly affected Group I participants by 45.2%, while it affected the control group by 37.3%. In-depth analysis by Perceived Stress Scale, the high perceived stress embodied in the crucial contrast amongst the groups (20.1% in Group I vs. 10.3% in Group II). In addition, Group I was significantly exemplified by the lack of physical activity as well as consumption of high-fat, processed, and fast food (33.7% in Group 1 vs. 27.1% in Group II). Furthermore, Group I suffered from notable low income in comparison with Group II.

**Table 1:** Basal characteristics, and risk factors of all patients in both groups.


	GROUP I (N = 840) (CVD GROUP)	GROUP II (N = 860) (CONTROL GROUP)	P VALUE

Age, years	55.61 ± 11.2	54.78 ± 9.12	0.094

Male gender, n (%)	436 (51.9%)	429 (49.9%)	0.405

Hypertension, n (%)	298 (35.5%)	262 (30.5%)	0.028*

Controlled hypertension, n (%)	108 (12.9%)	122 (14.2%)	0.423

Diabetes mellitus, n (%)	239 (28.5%)	208 (24.2%)	0.046*

Controlled diabetes mellitus, n (%)	155 (18.5%)	177 (20.6%)	0.268

Dyslipidemia, n (%)	311 (37.0%)	272 (31.6%)	0.019*

Obstructive sleep apnea, n (%)	26 (3.1%)	30 (3.5%)	0.650

Smoking, n (%)	186 (22.1%)	156 (18.1%)	0.040*

Atrial fibrillation, n (%)	61 (7.3%)	44 (5.1%)	0.066

Ischemic heart diseases, n (%)	599 (71.3%)	0 (0.0%)	0.001*

Cerebrovascular diseases, n (%)	203 (24.2%)	0 (0.0%)	0.001*

Peripheral arterial diseases, n (%)	224 (26.7%)	0 (0.0%)	0.001*

Chronic kidney diseases, n (%)	41 (4.9%)	43 (5.0%)	0.910

Family history of IHD, n (%)	80 (9.5%)	89 (10.3%)	0.570

Obesity, n (%)	277 (33.0%)	243 (28.3%)	0.035*

Anti-hypertensive medication use, n (%)	240 (28.6%)	237 (27.6%)	0.642

Cholesterol-lowering medication use, n (%)	283 (33.7%)	254 (29.5%)	0.065

Anti-platelet medication use, n (%)	317 (37.7%)	306 (35.6%)	0.356

Metabolic syndrome, n (%)	296 (35.2%)	242 (28.1%)	0.002*


*: significant P value, IHD; ischemic heart disease.

**Table 2:** Socioeconomic factors of all patients in both groups.


	GROUP I (N = 840) (CVD GROUP)	GROUP II (N = 860) (CONTROL GROUP)	P VALUE

Psychosocial stress, n (%)	380 (45.2%)	321 (37.3%)	0.001*

Perceived Stress Scale			

0–13: low stress, n (%)	86 (10.2%)	110 (12.8%)	0.099

14–26: moderate stress, n (%)	125 (14.9%)	122 (14.2%)	0.684

27–40: high perceived stress, n (%)	169 (20.1%)	89 (10.3%)	0.001*

Inadequate sleep, n (%)	110 (13.1%)	125 (14.5%)	0.390

Lack of physical activity, n (%)	387 (46.1%)	351 (40.8%)	0.029*

Dietary habits			

Excess sugar-sweetened beverages/day, n (%)	325 (28.0%)	224 (26.0%)	0.370

Excess salt intake, n (%)	202 (24.0%)	190 (22.1%)	0.339

Adequate fruits and vegetables/day, n (%)	146 (17.4%)	160 (18.6%)	0.511

High-fat, processed, and fast food, n (%)	283 (33.7%)	233 (27.1%)	0.003*

Marital status			

Married, n (%)	611 (72.7%)	648 (75.3%)	0.220

Separated/Divorced/Single, n (%)	229 (27.3%)	212 (24.7%)	

Income category			

High income, n (%)	325 (38.7%)	379 (44.1%)	0.024*

Low income, n (%)	515 (61.3%)	481 (55.9%)	

Level of education			

Bachelor’s degree or higher, n (%)	396 (47.1%)	444 (51.6%)	0.064

High school or less, n (%)	444 (52.9%)	416 (48.4%)	

Residence			

Urban, n (%)	362 (43.1%)	384 (44.7%)	0.518

Rural, n (%)	478 (56.9%)	476 (55.3%)	

Social isolation			

Lives alone, n (%)	81 (9.6%)	78 (9.1%)	0.685

Lives with others, n (%)	759 (90.4%)	782 (90.9%)	

Occupational status,			

Employed, n (%)	406 (48.3%)	405 (47.1%)	0.609

Unemployed, n (%)	434 (51.7%)	455 (52.9%)	

Health insurance, n (%)	426 (50.7%)	460 (53.5%)	0.252

Regular visits of CVD screening clinics, n (%)	332 (39.5%)	310 (36.0%)	0.139

Compliance with medical treatment, n (%)	369 (43.9%)	378 (44.0%)	0.992


*: significant P value, CVD; cardiovascular diseases.

As regards the physical assessment of all patients in both groups, Table 3 showcases substantial distinctions in the manner of greater body mass index in Group I than in Group II (26.5 ± 4.44 vs. 25.8 ± 4.40, P = 0.003) and waist circumference (99.02 ± 8.73 vs. 98.14 ± 8.43, P = 0.035). Concerning the laboratory findings, glycated haemoglobin (HbA1c) raised substantially in Group I (6.61 ± 1.51 vs. 6.48 ± 1.22, P = 0.048) however, the plasma glucose level 2-h postprandial and fasting plasma glucose level showed subtle differences between the two groups. The discrepancies in lipid profiles between the two groups were insignificant aside from low-density lipoprotein (LDL) cholesterol levels were higher in Group I than Group II (135.0 ± 25.1 mg/dl vs.132.4 ± 25.5 mg/dl, P = 0.033).

**Table 3:** Physical and laboratory assessment of all patients in both groups.


	GROUP I (N = 840) (CVD GROUP)	GROUP II (N = 860) (CONTROL GROUP)	P VALUE

Body mass index, (kg/m2)	26.53 ± 4.44	25.88 ± 4.40	0.003*

Waist circumference, (cm)	99.02 ± 8.73	98.14 ± 8.43	0.035*

Systolic BP, mmHg	133.0 ± 12.5	131.9 ± 12.8	0.072

Diastolic BP, mmHg	78.04 ± 10.2	77.86 ± 10.5	0.717

Heart rate, bpm	76.31 ± 12.9	75.61 ± 12.6	0.262

Fasting plasma glucose (mg/dl)	112.3 ± 9.21	111.8 ± 10.2	0.298

2-h post prandial plasma glucose (mg/dl)	146.2 ± 24.6	144.9 ± 23.4	0.271

HbA1c %	6.61 ± 1.51	6.48 ± 1.22	0.048*

Hemoglobin, g/dl	12.3 ± 0.85	1.2.2 ± 0.88	0.076

Thyroid Stimulating Hormone (mlU/L)	4.22 ± 1.76	4.33 ± 1.91	0.200

Total cholesterol, mg/dl	213.2 ± 37.3	211.9 ± 33.9	0.456

Serum triglycerides,mg/dl	154.3 ± 12.0	153.3 ± 12.6	0.087

Low density lipoprotein,mg/dl	135.0 ± 25.1	132.4 ± 25.5	0.033*

High density lipoprotein,mg/dl	44.1 ± 7.11	44.8 ± 7.16	0.068

Serum creatinine, mg/dl	1.08 ± 0.26	1.06 ± 0.25	0.215

e-GFR (mL/min/1.73 m2)	90.3 ± 10.9	91.1 ± 11.8	0.113

C-reactive protein,mg/L	4.42 ± 1.61	4.30 ± 1.64	0.137

Uric acid,mg/dl	5.79 ± 0.97	5.82 ± 0.96	0.613

Albuminuria (mg/g)	21.9 ± 8.90	21.4 ± 24.4	0.588


*: significant P value, BP: blood pressure, HbA1c: glycated hemoglobin, e-GFR: estimated glomerular filtration rate.

**Figure 1:**
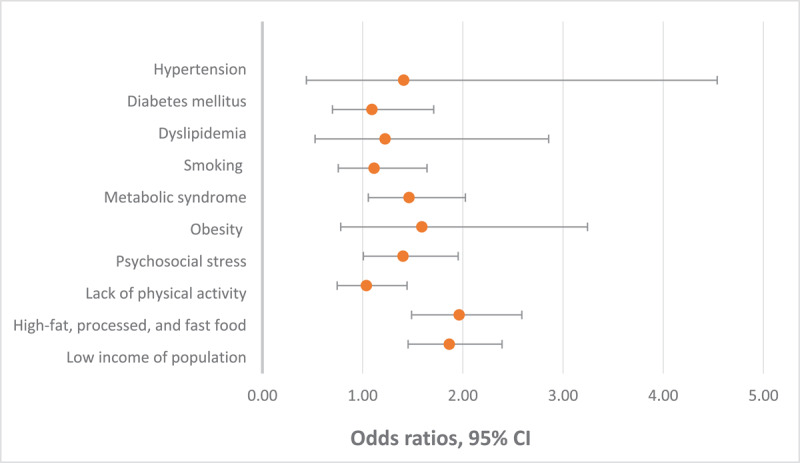
Forest plot of the multivariate regression analysis showing odds ratios and 95% confidence interval (CI) of the independent predictors affecting cardiovascular diseases.

Multivariate regression analysis identified the determining risk factors that impacted cardiovascular diseases in the CVD group, which were the existence of metabolic syndrome (OR = 1.463; 95% CI, 1.056–2.026; P = 0.022), the presence of psychosocial stress (OR = 1.404; 95% CI, 1.008–1.953; P = 0.044), the excessive intake of high-fat, processed, and fast food (OR = 1.964; 95% CI, 1.489–2.590; P = 0.001), and decreased population income (OR = 1.865; 95% CI, 1.454–2.391; P = 0.001), as illustrated in Table 4 and Figure 1.

**Table 4:** Multivariate regression analysis showing the independent predictors affecting cardiovascular diseases.


	MULTIVARIATE ANALYSIS		P. VALUE

	OR	(95% CI)	

Hypertension	1.410	0.438–4.539	0.565

Diabetes mellitus	1.093	0.699–1.710	0.697

Dyslipidemia	1.225	0.525–2.857	0.639

Smoking	1.115	0.756–1.643	0.583

Metabolic syndrome	1.463	1.056–2.026	0.022*

Obesity	1.592	0.781–3.245	0.201

Psychosocial stress	1.404	1.008–1.953	0.044*

Lack of physical activity	1.038	0.746–1.444	0.825

High-fat, processed, and fast food	1.964	1.489–2.590	0.001*

Low income of population	1.865	1.454–2.391	0.001*


*: significant P value.

## Discussion

The leading cause of mortality and morbidity on a worldwide scale is ACVDs. A considerable portion of the burden has been borne by low- and middle-income nations, as the morbidity associated with cardiovascular disease has amplified significantly over the past two decades (17). The etiology of ACVDs is multifactorial, and its clinical manifestations are heterogeneous. The biological nature of each individual with atherosclerosis is distinctive, resulting from the cumulative and continuous effects of genotype, phenotypic expression, and interaction with the environment. A multitude of pathways leads to diverse clinical manifestations, with hundreds of proteins and genes implicated in each stage of the atherosclerotic process (18).

Despite the irrelevant variance in both age and sex among the two groups in our results, the prevalence of DM, metabolic syndrome, and obesity was significantly higher in Group I, demonstrating the major basal features and risk reasons preceding ACVDs. In diabetic individuals, atherogenesis is a result of a variety of factors; insulin resistance and elevated lipid levels, which are prevalent in diabetes, are the primary catalysts of atherogenic injury. The endothelium in diabetic arteries is also believed to be more susceptible to atherogenic injury as a result of the decreased production of endothelial nitric oxide, which is known to be anti-atherogenic, and the increased production of plasminogen activator inhibitors (19). In addition, Type 2 diabetes mellitus (T2DM) is characterized by a complex combination of mechanistic processes, including oxidative stress, augmented homeostatic activation, abnormal vascular reactivity, renal dysfunction, and enhanced atherogenicity of cholesterol particles. These processes may increase the risk of CAD (20). In the estimation of ACVD risk in diabetic patients in the Middle East, the ratio was 19.6% in Egyptian diabetic patients, with 27% of them meeting the ESC criteria of very high risk and 72.1% being high risk (21).

Smoking and physical inactivity prevailed significantly among the CVD group as well as the incidence of HTN, DM, and dyslipidaemia especially higher levels of LDL. In line with our findings, Reiner et al. reported that patients with peripheral arterial diseases (PAD) who had been previously smoking markedly more often were less active physically, reported a previous diagnosis of DM more commonly, and had considerably higher rates of renal insufficiency, and higher blood pressure levels in comparison with patients without PAD (22). Additionally, their high-density lipoprotein-cholesterol (HDL-C) levels were markedly lower, and their serum triglycerides were markedly higher. The prevalence of risk factors of CAD, including physical inactivity, hypertension, obesity, hypercholesterolemia, smoking, diabetes mellitus, and low fruit and vegetable consumption, was 35.7%, 29.5%, 24.9%, 22.7%, 19.2%, 15.5%, and 90%, respectively regarding the stepwise survey piloted among the adult population aged 15–69 years (23). Higher levels of stress (20.1%) and bad dietary habits in the form of high intake of fast processed food (33.7%) were the characteristics of the CVD group. Abd El-Gawad et al. (24) showed that the most common risk factor for CAD was insomnia (73.5%), followed by carbonated beverage consumption (62.3%) and oily meat (46.4%). Additionally, stress (46.6%), overweight/obesity (34.2%), physical inactivity (34%), and hypertension (19.5%) were the most common risk factors.

Regarding stress as a risk factor, the Bahnasawy et al. study (25) also was in agreement with our results and reported that 69.03% of the CAD female patients were suffering from stress. The cardiovascular system is significantly affected by psychosocial stress, and a variety of cardiovascular diseases are influenced by stressors, particularly psychosocial, behavioural, and mental stressors (26). The release of the sympathomimetic hormone; catecholamine is an acute response, resulting in vasoconstriction and increased platelet activation, altering blood clotting, and thus accelerating the development of atherosclerosis (27). Significant peripheral arterial vasoconstriction is induced by mental stress, resulting in an increase in heart rate and blood pressure. Moreover, stress increases coagulation and the capability of blocking coronary arteries and leading to heart attack. Furthermore, the overproduction of free radicals, which pose a significant internal hazard to the cellular homeostasis of aerobic organisms, is a contributing factor (27). In this respect, Chandola et al. (28) suggested that CAD may be influenced by work stress through the neuroendocrine response activation to stressors directly or through detrimental behaviours that increase the risk of CAD, such as smoking, excessive alcohol consumption, or lack of physical activity indirectly. Gilbert-Ouimet et al. (29) stated that the increasing blood pressure of men and women who work long hours was linked to occupational stress. Through a variety of physiological mechanisms, physical inactivity may lead to CAD, which is partially attributed to the negative impacts on serum lipoprotein profiles, and blood pressure (30). Moreover, there was a noticeable association between the low-income population and CVDs significant in our study, as approximately 61.3% of the CVD group presented in the low-income category. In parallel, the time gap in performing primary catheter intervention and patients’ incapacity to remain on CVD guideline proper medications due to their expensive cost are the primary drivers of this problem in lower income population (31).

Some of the trends in IHD across various middle and low-income countries can be attributed to the increase in atherosclerotic risk factors. In addition, the health social determinants have a substantial effect on the underdiagnosis and underreporting of ACVDs in various regions. The past three decades had seen a variety of population-level policy reforms that had influenced IHD expansions by targeting risk factors and reducing ACVDs across countries, time, and regions (32). Furthermore, there is an increasing frame of evidence that an elevated risk of ACVDs is present prior to an acute ischemic event, and it is not uncommon for traditional risk factors to be identified prior to the occurrence of an acute ischemic event (33). As an example, impaired glucose tolerance is associated with a 20–30% increased risk of developing ACVDs in the absence of overt type 2 DM. This increase in risk is apparent with higher HbA1c in people within the so-called normoglycemic range (34).

Thomas et al. (35) found that IHD was the primary reason for the total global burden of ACVDs in 2016, with stroke following in second place, and that the majority of ACVDs had experienced an increase in total burden over time between 2000 and 2016, with the total burden of ACVDs attributable to each risk factor having increased. According to the current study, metabolic syndrome, psychosocial stress, high-fat, processed, and fast food, and poor income were associated with cardiovascular affection in Group I, despite the similarity in age and sex between the two groups. Notably, distinguishing from 1990, high systolic blood pressure and high LDL-C remain the uppermost two attributable risk factors in 2019, followed by high fasting blood glucose and high BMI (36). Therefore, hypertension is the most significant avoidable risk factor for CVD and all-cause death globally (37). Additionally, a substantial portion of the burden of preventable ACVDs is attributable to an unhealthy diet. In reality, the majority of ACVDs outcomes can be prevented by adopting a healthy diet and lifestyle, which includes a high intake of fruits and vegetables and a low intake of saturated fat, as well as exercising and quitting smoking (38,39,40).

## Conclusion

Atherosclerotic cardiovascular diseases have a life-threatening alarming burden, especially in low-income countries like Egypt. The prevalence of metabolic syndrome among Egyptians, the higher perceived psychosocial stress, the excessive consumption of high-fat, processed, and fast food, and the low income of the population were the main risk factors of ACVDs. Establishing a specific framework policy by gathering all the governmental and individual institutions exertions to intensify awareness towards preventive methods including proper control of diabetes, hypertension, and dyslipidemia, with encouraging healthy diet, physical exercise, and weight reduction and permitting all means to control ACVDs.

### Limitations

In order to demonstrate the imperative leading CVD risk factors, the current study’s outcome mostly rested on the control group, which was made up of the patient’s relatives and the visitors of outpatient clinics. This group shared many demographic and genetic characteristics with the group under investigation. It was a burden to extrapolate the findings to the whole Egyptian population due to the quite small sample size, which only denoted one demographic area. Further multicenter studies compulsory required to exemplify socioeconomic and metabolic avoidable risk, particularly in younger groups such as university students.

## Data Accessibility Statement

The datasets and materials analyzed in the current study are accessible from the corresponding author upon proper request.
